# Physical Activity Prescription in Primary Health Care: An Ethical Analysis

**DOI:** 10.3390/healthcare14070934

**Published:** 2026-04-03

**Authors:** Jesus Batuecas-Caletrio, Celia Álvarez-Bueno, Mar de Miguel Brox, Adrián Palacios-Diaz, María Frontelo-García, Beatriz Rodríguez-Martín

**Affiliations:** 1 Health Service of Castilla-La Mancha, Advanced Life Support Nursing, 45600 Talavera de la Reina, Spain; 2 Centro de Estudios Socio-Sanitarios, Grupo de investigación Age-ABC (Active Ageing, Health Behaviours and Cognition), Universidad de Castilla-La Mancha, 16071 Cuenca, Spain; celia.alvarezbueno@uclm.es (C.Á.-B.); mar.miguel@alu.uclm.es (M.d.M.B.); adrian.palacios1@alu.uclm.es (A.P.-D.); maria.frontelo@alu.uclm.es (M.F.-G.); beatriz.rmartin@uclm.es (B.R.-M.); 3 Facultad de Ciencias de la Salud, Universidad Autónoma de Chile, Talca 7500912, Chile; 4 Department of Nursing, Physiotherapy and Occupational Therapy, Faculty of Health Sciences, University of Castilla-La Mancha, Avda Real Fábrica de Sedas s/n, 45600 Talavera de la Reina, Spain

**Keywords:** primary health care, exercise therapy, ethics, clinical, health promotion, patient-centered care, patient autonomy, qualitative research

## Abstract

**Highlights:**

**What are the main findings?**

- Primary health care professionals perceive physical activity prescription as ethically demanding, involving ongoing negotiation between patient autonomy, professional responsibility, safety, and equity.
- Ethical tensions related to autonomy, beneficence, non-maleficence, and justice are addressed through context-sensitive strategies embedded in everyday clinical practice.

**What are the implications of the main findings?**

- Ethical physical activity prescription requires more than clinical knowledge; it depends on relational competence, ethical judgment, and supportive health system structures.
- Integrating ethical frameworks and organizational support mechanisms may strengthen the legitimacy, safety, and sustainability of physical activity promotion in primary health care.

**Abstract:**

**Background/Objectives**: Although prescribing physical activity (PA) is a well-established preventive strategy in primary health care (PHC), its ethical implications remain under-researched. This study examines how general practitioners (GPs) and nurses experience, interpret, and manage ethical tensions in PAP. **Methods**: A qualitative study was conducted with 28 PHC professionals (13 GPs, 15 nurses) from rural and urban centers in Toledo, Spain (*M* = 18.4 years of experience). Data were collected through semi-structured interviews and analyzed using reflexive thematic analysis. Beauchamp and Childress’ four-principles framework was applied abductively to synthesize ethical conflicts and coping strategies. **Results**: Two main themes emerged: (1) Ethical conflicts in PAP, characterized by tensions between autonomy and paternalism, and the challenge of balancing beneficence with non-maleficence under institutional pressures; and (2) Professional coping strategies, where clinicians used relational care, individualized tailoring, and interprofessional collaboration to mitigate moral distress. Results indicated that clinical codes, such as “unrealistic goals” or “institutional pressure,” often overlapped across multiple ethical principles, necessitating a nuanced, multi-dimensional approach to counseling. **Conclusions**: PAP is not a neutral clinical task but an ethically grounded practice constrained by structural and organizational factors. To move toward safe and equitable health promotion, PAP must be conceptualized as a relational intervention. We propose an Ethical Reflective Tool and a conceptual framework to support clinical reflection, enhance professional accountability, and guide policy-level support for preventive care in PHC.

## 1. Introduction

Physical activity (PA) has evolved from a lifestyle recommendation to a core clinical intervention within primary health care (PHC). As a central pillar of preventive medicine, physical activity prescription (PAP) is now recognized as having therapeutic efficacy comparable to pharmacological interventions in the management of coronary heart disease, stroke rehabilitation, and diabetes prevention [1]. Beyond physical health, recent evidence highlights its role in reducing dementia risk and improving mental health outcomes [2]. Consequently, general practitioners (GPs) and nurses are increasingly tasked with integrating PA counseling into routine consultations, often utilizing structured frameworks like the “5A’s” model (Assess, Advise, Agree, Assist, Arrange) to ensure individualized implementation [3,4]. In this context, PAP is not merely a biomedical instruction but a complex health-promotion practice that functions as the first point of contact between community health and the healthcare system.

While the clinical benefits of PA are well-established, the act of prescribing it is a morally charged intervention that transcends simple medical advice. Ethics in this field serves as a philosophical reflection on the “right” action, promoting values that must adapt to diverse clinical circumstances [5,6]. Within the PHC setting, these actions are best understood through Beauchamp and Childress’ four-principles framework: autonomy (respecting the patient’s right to choose), beneficence (acting in the patient’s best interest), non-maleficence (avoiding harm or undue pressure), and justice (ensuring equitable access to health resources) [7]. Ethical PAP requires a constant negotiation between these principles, particularly when professional responsibilities to promote health intersect with the patient’s personal values and socio-cultural context [8].

The ethical dimensions of lifestyle counseling have been explored broadly in relation to chronic disease management and shared decision-making [9]. Previous literature indicates that while core concepts like informed consent and confidentiality are well-integrated into medical education, the specific ethical tensions inherent in lifestyle interventions—such as the risk of paternalism or the impact of social determinants on a patient’s ability to follow a prescription—remain underexplored [10]. Studies in related fields, such as nutritional counseling and smoking cessation, suggest that clinicians often face dilemmas regarding patient “compliance” versus “choice,” yet few studies have specifically mapped these tensions within the unique longitudinal relationship of PHC and PA [11,12]. Furthermore, external influences and institutional pressures are known to affect prescribing behaviors, potentially compromising the ethical standards of individualized care [13].

Despite the growing prevalence of PAP, there remains a significant gap in understanding how front-line clinicians experience and navigate these ethical dilemmas in daily practice. Current literature lacks a nuanced exploration of the specific tensions felt by GPs and nurses when balancing the duty to promote activity against the realities of patient barriers and systemic constraints. This study addresses this gap by qualitatively exploring how PHC professionals perceive and manage the ethical dimensions of prescribing PA. Using the four-principles framework as an analytical lens [7], we seek to identify the primary ethical conflicts encountered in the Spanish PHC context and provide a foundation for developing ethically sound, equitable practices in health promotion.

## 2. Materials and Methods

### 2.1. Design and Participants

This study employed a qualitative descriptive design to explore primary health care (PHC) professionals’ experiences with physical activity prescription (PAP) and associated ethical dilemmas [14,15]. The study adhered to the Consolidated Criteria for Reporting Qualitative Research (COREQ) [16].

A total of 28 participants (13 GPs and 15 nurses) were recruited from rural and urban primary health care (PHC) centers in Toledo, Spain, using an intentional theoretical sampling strategy. In this region, “GPs” include specialists in Family and Community Medicine. We ensured a diverse range of professional statuses. To minimize selection bias, invitations were extended to all professionals in the health centers via professional mailing lists, and brief in-person briefings during clinical staff meetings, followed by phone calls and snowball sampling. Inclusion criteria required active clinical practice and experience in lifestyle counseling. Exclusion criteria included those on long-term leave, retired, or unable to communicate in Spanish. To ensure transparency, we recruited both early-career and senior specialists with over 20 years of experience (see Table 1).

### 2.2. Data Collection and Data Sources

Semi-structured interviews (average 90 min) were conducted face-to-face in private settings and provided “naive descriptions” of the phenomenon in the participants’ own words [15,17]. All interviews were audio-recorded and transcribed verbatim by the research team to ensure linguistic precision. The interview guide (Table 2) was iteratively refined using the constant comparative method, incorporating insights gained from successive interviews [18].

Data collection and analysis were conducted concurrently. Saturation was operationalized through the “code saturation” approach [19]; after the 23rd interview, new data confirmed existing categories without generating new themes. An additional five interviews were conducted to ensure “meaning saturation,” confirming that the conceptual dimensions of ethical tensions (e.g., the conflict between patient autonomy and clinical beneficence) were fully explored and no further nuances emerged.

### 2.3. Analysis

Data were analyzed using the six-step reflexive thematic analysis proposed by Braun and Clarke (2006) [20]: (1) Familiarization with the data; (2) Systematic coding; (3) Generation of themes; (4) Reviewing themes; (5) Refining and naming themes; and (6) Report writing. To address the risk of “forcing” data into pre-existing categories, the coding process was primarily inductive. Two trained researchers independently coded the first five transcripts to develop an initial codebook. Disagreements were resolved through peer-debriefing and consensus meetings. Only during the final thematic synthesis were the themes interpreted through the lens of Beauchamp and Childress’ four-principles framework [7] to support conceptual depth. Transcripts were managed using ATLAS.ti 25 software (ATLAS.ti Scientific Software Development GmbH, Berlin, Germany). To ensure a concise presentation of results while maintaining evidence-based depth, full-length transcripts and expanded verbatims are available in the Supplementary Files associated with this study.

### 2.4. Trustworthiness and Ethics

To ensure rigor, we applied the criteria of credibility, transferability, dependability, and confirmability [21]. Several strategies as researcher triangulation and participant validation were implemented. Reflexivity was maintained via a reflexive journal. For example, the lead researcher (a nurse-anthropologist) documented how her “outsider” status as a non-clinician in the Toledo region allowed her to probe “obvious” clinical routines that a peer might have overlooked. Furthermore, during coding, the team specifically discussed cultural and linguistic nuances, such as the Spanish concept of ‘confianza’ (trust), ensuring these relational values were not lost when mapped to the four universal principles.

Ethics Statement: This study was approved by the Ethics and Research Committee of the Health Integrated Area of Talavera de la Reina (Ref: SBPLY/17/180501/000533). All participants provided written informed consent, and confidentiality was maintained through anonymized coding (e.g., 01FnurseUrb).

## 3. Results

Primary health care professionals faced significant ethical conflicts when prescribing PA, requiring various strategies to navigate these tensions. Consequently, two main themes were identified: (1) Ethical conflicts in PA prescription, analyzed through the four-principles framework; and (2) Professional coping strategies. Table 3 provides a comprehensive selection of representative verbatims and associated clinical codes for the first theme, while Table 4 maps the multi-dimensional nature of the identified ethical tensions, illustrating the conceptual overlaps between the four ethical principles and the corresponding clinical codes. Finally, Table 5 presents the verbatims and codes associated with professional coping strategies. All quotes are identified by the interview number, gender (F: female and M: male), participant profession (GP or nurse), and setting (Urban or Rural).

Due to the depth of the qualitative data, the tables in this manuscript present a summarized selection of the most representative quotes. A comprehensive set of full-length verbatims for all themes and sub-themes is provided as Supplementary Materials (Tables S1 and S2).

### 3.1. Theme 1: Ethical Conflicts in Physical Activity Prescription (PAP)

Participants identified diverse ethical tensions emerging from the intersection of clinical duty, patient reality, and systemic constraints. These were analyzed through the lens of Beauchamp and Childress’ framework, alongside emerging emotional and institutional barriers.

#### 3.1.1. Autonomy: Paternalism, Culture, and Resistance

A primary tension existed between supporting patient autonomy and falling into paternalistic “motherly” roles, where clinicians felt they “must” direct the patient rather than empower them. Success in PAP was seen as dependent on moving from directive advice to collaborative goal-setting. However, autonomy was often bounded by socio-cultural determinants. Participants noted that certain ethnic groups or family structures perceived as less engaged in self-care presented barriers that felt “stronger than therapeutic efforts”. Additionally, clinicians had to navigate the ethics of patient refusal or indifference, where the professional duty to promote health reached a limit against a patient’s right to remain sedentary.

#### 3.1.2. Beneficence: Competence and Holistic Care

Beneficence was challenged by a perceived lack of professional competence. Many clinicians felt limited to an advisory role because they lacked formal training in structured exercise physiology, which they felt undermined their ability to support genuine behavior change. Furthermore, “acting in the best interest” required a holistic perspective. Prescribing PA without considering comorbidities (e.g., knee pain) or social context (work–life balance) was viewed as potentially harmful, risking patient rejection of PA altogether. These efforts were further undermined by institutional pressures that prioritized patient quantity over the quality of the therapeutic relationship.

#### 3.1.3. Non-Maleficence: Safety, Blame, and Emotional Harm

Concerns regarding non-maleficence centered on the fear of legal responsibility for adverse events, such as cardiac strain or falls. This “defensive medicine” mindset created uncertainty in recommending vigorous activity. Beyond physical safety, clinicians noted the risk of emotional harm. Many patients minimized their inactivity or justified daily chores as exercise out of embarrassment or shame. This created a “vicious cycle” where the clinician had to address the ethical weight of the patient’s feelings of failure regarding weight and pain.

#### 3.1.4. Justice: Geographic and Systemic Equity

Justice-related conflicts emerged primarily from geographical inequities. Rural areas offered natural spaces but lacked structured facilities for younger populations, whereas urban environments provided gyms but could be more restrictive for outdoor walking.

#### 3.1.5. Emotional Barriers and Professional Moral Distress

Finally, a significant “relational” ethical conflict existed in the incoherence between professional advice and personal behavior. Clinicians expressed that being sedentary or obese while prescribing PA undermined their credibility and caused personal ethical discomfort. This was exacerbated by a lack of workplace support, where the absence of staffing substitutes or technological resources forced clinicians to prepare health education on their own time, leading to professional burnout and moral distress.

#### 3.1.6. Synthesis of Ethical Multi-Dimensionality

While the four principles of Beauchamp and Childress provided a robust structural framework for analysis, the narratives revealed that many ethical dilemmas were inherently multi-dimensional and resistant to single-category classification. For example, the code ‘Unrealistic Goals’ simultaneously challenged Beneficence (by failing to achieve the intended health improvement) and Non-maleficence (by risking physical injury, such as joint damage). Similarly, ‘Institutional Pressure’ acted as a systemic barrier to both Beneficence (limiting the quality of care) and Justice (affecting the equitable allocation of clinical time). Table 4 illustrates these conceptual overlaps, reflecting the complex, non-linear nature of ethical decision-making in primary health care.

### 3.2. Theme 2: Coping Strategies for Navigating Ethical Conflicts in PAP

To manage the ethical tensions described in Theme 1, participants employed four primary categories of coping strategies. These strategies move PAP from a top-down medical instruction to a relational, ethically grounded process.

#### 3.2.1. Patient-Centered Relational Ethics

The most prominent strategy involved validating relational autonomy through active listening and tailored assessment. Clinicians rejected standardized advice in favor of “sustainable” plans that integrated with the patient’s social and family life. By offering inclusive options—such as dancing or walking outdoors—professionals shifted the ethical focus from “compliance” to “enjoyment,” which they perceived as essential for long-term adherence.

#### 3.2.2. Continuity, Documentation, and Accountability

To uphold the principles of beneficence and non-maleficence, clinicians emphasized the need for a “structured physical activity plan” rather than informal advice. Long-term follow-up was viewed as a moral safeguard; without it, professionals felt their advice was “useless” or potentially unsafe. Furthermore, using documentation and electronic records served to legitimize PAP as a formal clinical intervention, providing a “paper trail” that supported institutional accountability and justified the time spent on prevention.

#### 3.2.3. Professional Credibility and Role Modeling

Clinicians identified personal experience and “leading by example” as vital for ethical credibility. There was a strong consensus that an “unhealthy” or sedentary professional undermines the ethical weight of the prescription. Conversely, professional recognition—such as public acknowledgement or social reinforcement from managers—acted as a powerful motivator to sustain these health-promotion efforts despite high workloads.

#### 3.2.4. Shared Ethical Responsibility and Collaboration

Finally, participants highlighted interprofessional collaboration as a strategy to address the “Justice” and “Competence” gaps. By sharing the care pathway—where physicians provided the initial clinical indication and nurses coordinated the 15-day follow-up—the team ensured the prescription was safe and feasible. This collective approach mitigated the burden on individual clinicians and embedded PAP into the official service portfolio, making it a “truly successful treatment” rather than a fragmented effort.

**Figure 1:**
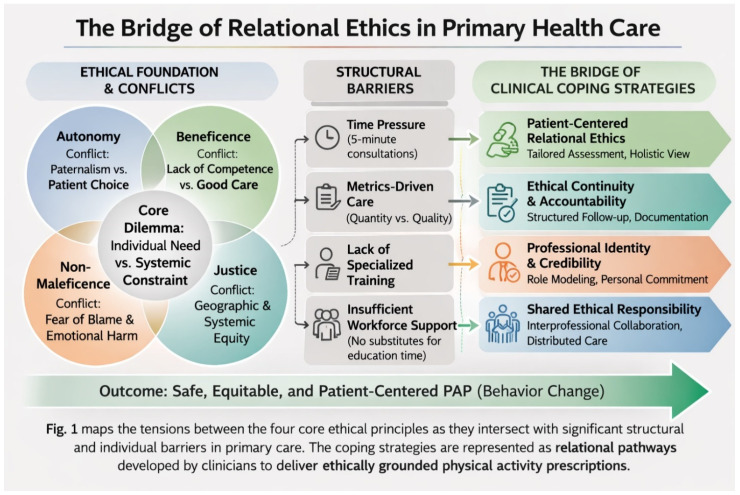
Conceptual framework of the ethical landscape of PAP, illustrating the tensions between ethical principles, structural barriers, and professional coping strategies.

The dynamic relationship between these identified ethical conflicts, the structural barriers of the health system, and the practical coping strategies employed by clinicians is synthesized in Figure 1. This conceptual framework illustrates how professionals navigate institutional constraints to realign their practice with ethical principles:

## 4. Discussion

This study explores physical activity prescription (PAP) from an ethical perspective, illustrating how primary health care (PHC) professionals manage ethical tensions in daily practice. Findings demonstrate that PAP is a morally complex intervention shaped by relational care, professional responsibility, and structural constraints. Ethical principles—autonomy, beneficence, non-maleficence, and justice—are not abstract concepts but are enacted within specific organizational and social contexts.

### 4.1. Navigating Ethical Tensions in Health Promotion

The ethical tensions identified in PAP mirror broader challenges in preventive care, such as obesity management, where clinicians balance the duty to promote health (beneficence) with the risk of paternalism [22]. This struggle between directive guidance and respecting choice is central to shared decision-making literature [23,24]. Systemically, institutional pressures often prioritize quantitative targets over the relational time required for person-centered behavior change [25,26]. In this study, autonomy emerged as relational rather than purely individual, with trust-building, active listening, and negotiated goal-setting serving as core ethical practices [27]. Professionals must navigate cultural and family dynamics without moralizing behaviors [28,29]. Accepting a patient’s refusal is ethically demanding but necessary to uphold voluntary choice [7,30].

Beneficence was reflected in the commitment to safe and meaningful PA. However, this intent was often constrained by gaps in professional training and confidence [31]. This highlights a system-level responsibility to ensure competence as a core principle of beneficent care [32]. Additionally, organizational pressures—such as limited consultation time—undermine the capacity for personalized care, placing clinicians in conflict with institutional efficiency metrics [33,34].

Regarding non-maleficence, concerns about physical injury and legal accountability were prominent. Fear of blame often leads to conservative recommendations to avoid potential harm [35]. At the same time, participants’ focus on supervision and follow-up aligns with evidence linking unsupervised interventions to lower adherence and higher attrition [36]. Beyond physical risks, clinicians identified the risk of emotional harm, such as patient embarrassment or disengagement. Preserving dignity through non-stigmatizing communication is essential for ethical PAP [37].

Justice emerged through the lens of unequal access to resources. Geographic disparities between rural and urban settings reflect structural inequities [38]. Prescribing PA without acknowledging these constraints risks reinforcing health inequities. Therefore, PHC professionals must act as advocates for equitable access to safe and affordable PA [39].

### 4.2. Institutional Pressures and Moral Distress

A significant finding was the impact of institutional constraints on ethical practice. Productivity-driven metrics prioritize “quantity over quality,” marginalizing the relational time needed for effective counseling [40]. This creates moral distress, where clinicians know the “right” course of action (e.g., a 20 min motivational interview) but are prevented from doing so by a 5 min consultation window [41].

Furthermore, our findings on role modeling suggest that a clinician’s personal health behavior is not just a personal choice but an ethical asset. Incoherence between advice and behavior undermines professional credibility, a phenomenon also observed in obesity management and alcohol counseling [42].

### 4.3. Strategies as Ethical Coping Mechanisms and Practical Implications

The strategies identified in this study function as practical responses to ethical conflicts, enabling clinicians to realign PAP with ethical principles despite real-world constraints. These coping mechanisms provide a foundation for operationalizing PAP at clinical, institutional, and policy levels:

- Clinical Level: Relational and Individualized Care. Strategies such as comprehensive assessment, individualized tailoring, and active listening operationalize the principles of beneficence and autonomy. By offering inclusive options and explaining purposes/risks, clinicians mirror patient-centered approaches that strengthen trust and shared responsibility [43]. To support this, we propose the Ethical Reflective Tool for Clinical Practice (Appendix A), which could be integrated into Electronic Medical Records (EMR). This tool reduces cognitive load by prompting specific questions on social determinants (Justice), standardizing co-morbidity assessments (Non-maleficence), and documenting the “Agree” and “Assist” stages of the 5As model.
- Institutional Level: Accountability and Collaboration. Documentation serves as both an ethical and organizational mechanism, supporting accountability and the recognition of preventive work [44]. Interprofessional collaboration further advances justice by distributing responsibility across the care team—for example, with GPs providing clinical indications while nurses coordinate follow-up. This shared accountability mitigates individual “fear of blame” and optimizes complementary expertise [43,45].
- Policy Level: Support and Professional Identity. Professional recognition and role modeling emerge as ethical reinforcers that mitigate moral distress and sustain engagement [46]. Strengthening education in exercise prescription and protecting “relational time” within clinical agendas are essential policies to reduce ethical tension. Ultimately, PAP must be conceptualized as an ethically grounded component of PHC that requires structural support to remain equitable and sustainable.

### 4.4. Strengths, Limitations, and Future Research

A key strength of this study is its robust ethical framing—grounded in the ICN Code of Ethics [32] and Beauchamp and Childress’ principles—which facilitated a deep, contextualized exploration of physical activity prescription as a morally grounded practice. However, several limitations must be acknowledged. Findings are situated within a specific Spanish primary care context, potentially limiting transferability, and the reliance on self-reported data may introduce social desirability bias. Furthermore, while the Ethical Reflective Tool for Clinical Practice (Appendix A) serves as a structured tool for clinical reflection, it is intended as a practical synthesis based directly on the participants’ shared strategies, rather than a formally validated instrument. It has not undergone formal psychometric validation. Future research should prioritize the methodological validation of this checklist to ensure its applicability across diverse healthcare settings and incorporate patient perspectives to further refine this relational ethical framework.

Furthermore, the absence of patient and interprofessional perspectives restricted a holistic view of ethical dynamics. Crucially, future research should incorporate patient voices to understand their lived experiences of clinician paternalism or the emotional impact of sedentary behavior. Comparative studies across diverse health systems (e.g., those with versus without exercise referral schemes) would also further illuminate the structural determinants of ethical PAP and support a more comprehensive relational ethical framework.

## 5. Conclusions

This study demonstrates that PAP is an ethically complex intervention that requires general practitioners and nurses to navigate constant tensions between Beauchamp and Childress’ four core principles. Our findings reveal that ethical dilemmas in PAP are not merely individual clinical hurdles but are deeply rooted in structural barriers, such as time scarcity and a lack of specialized training. To move from “informal advice” to an ethically grounded clinical practice, health systems must transition toward a relational ethics model. This includes:

- Tailored Guidance: Moving beyond paternalistic “motherly” roles to support patient empowerment and cultural diversity.
- Institutional Support: Recognizing PAP as a legitimate, time-intensive clinical task that requires interprofessional collaboration and integrated documentation.
- Practical Tools: Implementing ethical reflection tools, such as the proposed “Ethical Reflective Tool for Clinical Practice” (Appendix A), to help clinicians manage moral distress and ensure equitable care.

By integrating ethical reflection into professional development and clinical policy, primary care systems can better support clinicians in navigating these challenges. Ultimately, fostering an environment that values preventive and relational care is essential to ensuring that physical activity promotion remains safe, equitable, and truly patient-centered.

## Figures and Tables

**Table 1:** Main characteristics of participants (N = 28).

Characteristics	Category	*n*	%
Profession	General Practitioner (Family Medicine Specialist)	13	46.4%
	Nurse	15	53.6%
Gender	Male	13	46.4%
	Female	15	53.6%
Education Level	Bachelor’s degree	9	32.1%
	Specialist (Post-graduate training)	5	17.9%
	Master’s Degree	9	32.1%
	PhD	5	17.9%
Work Setting	Urban	17	60.7%
	Rural	11	39.3%
Age (Years)	Mean (Range)	46.8 (28–64)	-
Experience in PHC	Mean (Years)	18.4	-

**Table 2:** Interview topic script.

Stage	Subject	Content
Introduction	Motives, reasons Ethical issues	I would like to know your experience with physical activity prescription or counseling in primary health care. Would you like to take part? Information about recording consent, the possibility of dropping out, and confidentiality.
Beginning	Introductory question	Tell me about what you mean by physical activity.
Development	Conversation guide	Own experience prescribing physical activity in primary health care. Ethical conflicts or dilemmas perceived when prescribing physical activity. Coping strategies used to deal with conflicts, refusals or patients preferences. Impact of ethical conflicts on your profession and on your day-to-day life as a health professional.
Closing	Final question Appreciation	Is there anything else you would like to tell me? Thank you very much for taking part. Your testimony is of great use for our research.

**Table 3:** Verbatims of Ethical conflicts in PA prescription (Theme 1).

Sub-Theme	Code	Verbatim
Autonomy	Paternalism	“We often take on a ‘motherly’ role. The person isn’t here to be told what to do; they need to find their own motivation through a sincere relationship.” (18FnurseUrb)
	Social/Cultural Barriers	“When you come across ethnic groups [less] inclined toward self-care, social barriers are stronger than your therapeutic approach.” (12MGPUrb)
	Refusal & Indifference	“Some don’t care: ‘If I have to die, I’ll die.’ There comes a point where you say: ‘It’s your life, you decide,’ but don’t blame me later.” (08FGPUrb)
Beneficence	Lack of Competence	“A nurse is trained to diagnose sedentary behavior, but to reach WHO recommendations? We were simply never taught that.” (01FnurseUrb)
	Holistic Context	“You tell them to walk for an hour and you end up damaging their knees. You can’t prescribe without considering their social and physical context.” (05MnurseUrb)
	Institutional Pressure	“The system focuses on quantity, not quality. The more patients you see, the ‘better’ doctor you are considered. We are rewarded for numbers.” (22MGPUrb)
Non-maleficence	Defensive Medicine	“If you tell someone to run and they break a hip, they’ll blame you. Nowadays, it feels like we are blamed for every accident.” (13MGPUrb)
	Emotional Harm (Shame)	“When patients don’t recognize their inactivity, they justify it with daily tasks [shopping, stairs], reflecting a certain degree of embarrassment.” (06MGPUrb)
Justice	Rural vs. Urban Equity	“In rural areas, walking is easier, but urban areas have better sports facilities. Rural youth don’t have gyms that fit their interests.” (03FnurseRur)
Emotional Barriers	Lack of Support	“Management gives zero support. If you want to run a group session, you do it at home, on your own time, using your own computer.” (07FnurseRur)
	Role Modeling	“I’ve seen doctors tell patients to stop smoking while holding a cigar. If you tell someone to lose weight, you have to set an example.” (01FnurseUrb)

**Table 4:** Mapping of Ethical Tensions and Clinical Codes.

Code	Autonomy	Beneficence	Non- Maleficence	Justice	Illustrative Quote
Paternalism	X	x			“Taking on a motherly role vs. building motivation”
Institutional Pressure		X		x	“Rewarded for quantity, not quality”
Unrealistic Goals		X	x		“Damaging knees by ignoring pathologies”
Defensive Medicine		x	X		“Fear of being blamed for accidents leads to overly cautious advice”
Resource Access	x			X	“Rural trails vs. urban sports facilities”

Upper-case ‘X’ indicates a primary ethical tension; lower-case ‘x’ indicates a secondary or overlapping tension.

**Table 5:** Verbatims of Strategies to navigate Ethical conflicts (Theme 2).

Sub-Theme	Code	Verbatim
Patient-Centered Ethical Practice	Tailored Assessment	“It consists of explaining the benefits but also how they should do it […] evaluating their specific condition and abilities to ensure the prescription is detailed and effective.” (14FGPUrb)
	Sustainability	“Exercise must be compatible with the person’s life—their job, social, and family life. It’s not for one or three months; it’s a lifestyle habit that sustains health as long as it’s practiced.” (05 MnurseRur)
	Active Listening	“Encouraging patients to choose between several options makes them active participants in their treatment. It makes the plan more tailored to their preferences.” (16 FnurseRur)
	Inclusive Options	“It shouldn’t feel like a sacrifice or a burden; they should enjoy it—whether it’s dancing or swimming. Without enjoyment, there is no adherence.” (05 MnurseRur)
Ethical Continuity & Accountability	Follow-up & Documentation	“Ideally, there should be a follow-up to see if it’s being done. If you prescribe something but don’t know the outcome, the advice serves no purpose.” (18 FnurseUrb)
	Structured Planning	“We should focus on giving a structured plan, agreeing on realistic goals, and assessing how the patient perceives the value of the prescription.” (20 MnurseUrb)
Professional Identity & Credibility	Personal Experience	“Your personal perception of exercise influences how you convey it. If you come across as unhealthy, the credibility of your recommendation is called into question.” (16 FnurseRur)
	Professional Recognition	“We organized an event where the manager thanked staff with a certificate. They left saying: ‘I feel like coming back tomorrow to keep doing it.’ Social reinforcement has a huge impact.” (01 FnurseUrb)
Shared Ethical Responsibility	Interprofessional Collaboration	“The physician could make the prescription and the nurse monitor the program every 15 days. That system would make this a truly successful treatment.” (02 MGPRur)

## Data Availability

The data presented in this study are available on request from the corresponding author.

## Supplementary Materials

The following supporting information can be downloaded at: https://www.mdpi.com/article/10.3390/healthcare14070934/s1, Table S1. Expanded Verbatims for Theme 1: Ethical Conflicts; Table S2. Expanded Verbatims for Theme 2: Coping Strategies.

## Abbreviations

The following abbreviations are used in this manuscript:

PAP	Physical Activity Prescription
PA	Physical Activity
GP	General Practitioners
ICN	International Council of Nurses

## Appendix A

**Ethical Reflective Tool for Clinical Practice**
**(Alignment with the ICN Code of Ethics-2021 elements)**

1. **Respect for Autonomy**

- Have I provided clear, understandable information about benefits and risks?
- Has the patient freely agreed or declined without pressure?

*Element 1: Respect for human rights, dignity, autonomy, and informed decision-making*

2. **Individualised Assessment**

- Have I assessed the patient’s health status, physical conditions, goals, and risk factors?
- Have I used appropriate screening tools (e.g., FITT-Frequency, Intensity, Time, Type)?

*Elements 1 & 4: Respect for individuality and accountability for sound professional judgement*

3. **Beneficence**

- Is this recommendation aligned with the patient’s long-term health and quality of life?

*Element 2: Promotion of health, prevention of illness, and alleviation of suffering*

4. **Non-Maleficence**

- Is the activity safe for the patient, and have precautions/warning signs been explained?

*Elements 2 & 4: Obligation to prevent harm and practice safely within competence*

5. **Equity and Justice**

- Have I considered the patient’s social, economic, cultural, and environmental context to ensure recommendations are realistic and accessible?

*Element 3: Advocacy for social justice, equity, and access to care*

6. **Professional accountability**

- Am I acting within my scope of practice and seeking collaboration when needed?

*Element 4: Accountability, responsibility, and competence in practice*

7. **Cultural Respect**

- Have I offered culturally appropriate and inclusive activity options?

*Elements 1 & 3: Respect for diversity and culturally safe care*

8. **Documentation**

- Have I documented assessment, consent, recommendations, response, and follow-up?

*Element 4: Ethical accountability, transparency, and continuity*
