# Supplementary material for: Physical Activity Prescription in Primary Health Care: An Ethical Analysis
Source: Healthcare (Basel). 2026 Apr 3;14(7):934. doi: 10.3390/healthcare14070934 (PMC13073350; doi:10.3390/healthcare14070934)
Supplement: Supplementary file 1 [file healthcare-14-00934-s001.zip › healthcare-4179968 Table S1. Expanded Verbatims for Theme 1.pdf]

Table S1. Expanded Verbatims for Theme 1: Ethical Conflicts experienced by health care professionals when prescribing physical activity

Sub-themes	Codes	Verbatims
Autonomy	Paternalism	"I think that many times we take on a sort of motherly role, like "oh, don't do this, don't do that", and I think that's a mistake. In the end, the person isn't here to be told what to do, but if you build a good relationship with them, sit down and talk with them and make the effort to get to know them, and let them get to know you, they see that what you tell them is true, that you're sincere, and little by little you start introducing things in other ways, giving bits of advice until they finally set a goal for themselves, because they've found their own motivation" (18FnurseUrb).
	Cultural beliefs or preferences	"If the family of origin doesn't have strong motivation, for example, I'm not— it's not that I'm racist, not at all, but when you come across some ethnic groups that are particularly… not inclined toward personal self-care, it's impossible, no matter how hard you try, you just can't manage it, the social barriers they have imposed on them are stronger than your therapeutic approach, stronger than your attempts to take a positive approach" (12MGPUrb).
	Privacy	"They do not comply, when I have advised some patients to join a gym to lose weight and improve their health, I have encountered the issue that they feel intimidated by their physical condition" (25FnurseUrb).
	Refusals & indifference	"But then some don't care one way or the other, they just don't care about anything, you tell them, "You need to lose weight," and they say, "What for?" and you tell them, "you should stop smoking, it's bad for you," and they go, "well… if I have to die, I'll die" so there comes a point when there are also patients to whom you just say, "look, it's your life, you're the one who decides what to do with it, I can tell you that you need to do this, this, this, and this, if you want to do it, great, and if not, that's fine too, but I always tell them — if you don't, then don't come back later telling me that things are going wrong" (08FGPUrb).
Beneficence	Lack of competence	"A nurse is not trained for that, she is trained to discern whether a person is sedentary, somewhat active, or active, and to make the diagnosis, and to give advice, I mean, everyone at least knows the four basic things to recommend and when physical exercise is not indicated, but if what we really want is to reach the recommendations set by the WHO, which call for doing something more — not just daily life activities, but repetitive exercise that makes you break a sweat, that you do routinely and incorporate as part of your life, just like you brush your teeth in the morning, you know you have to do at least half an hour of physical exercise every day, then I no longer blame health care professionals, because poor things — we were simply never taught that" (01FnurseUrb).
	Unrealistic goals	"Well, here's an important point, and it's very important what I'm about to say, and you know it, not only for the exercise to be effective but also so that it isn't harmful. Because many times the way exercise is prescribed ends up being counterproductive, you have to look at the person as a whole, a person who has knee problems, but also high blood pressure or high blood sugar. You tell them to walk for an hour, and you end up damaging their knees, okay? You can't make a prescription without considering all their pathologies, everything about that person. in their context, not just physically or pathologically, but also socially, in general, including family issues, it has to be compatible with their life, compatible with their work, otherwise it ends up causing rejection, or they end up thinking that exercise isn't good, or they abandon it for a thousand reasons because it doesn't fit into that person's life, either physically, socially, professionally, or within their family" (05MnurseUrb).
	Institutional pressure	"But being able to establish a relationship with the patient and being able to talk with them, and to take your time, that is, with the current system, the focus is not on providing quality care, instead, the more patients you see, the better a doctor you are considered, the more incentives you get, in other words, you're rewarded for quantity, not quality, but we've been suffering from that for a long time" (22MGPUrb).
		"The people I've told, either I haven't been able to follow up to see what happened afterward, because, as I said, maybe I was covering for a week or two, or just a few days in primary care, and of course,

Non-maleficence	Lack of follow-up responsibility	once you leave, you never know what happens next, or because the people I've told have been a bit reluctant, saying things like, "oh, I don't want to sign up for a gym" or "I don't really feel like going out for a walk". I mean, usually the people who do go out walking are the typical ladies who come to the health center, and they already have their routine of going out walking with neighbors or friends, which is great, but if it's people who are already sedentary by nature, when you tell them to do physical exercise or something like that, they tend to be reluctant at first, either because they say they don't have time, or because they don't really find much enjoyment in doing it" (03FnurseRur).
	Defensive Medicine	"The issue of defensive medicine, when you prescribe exercise to someone, you have to take on the possible negative consequences, like accidents, you know? Yes, if you tell someone to go running, and they fall while running and break a hip, they'll blame you (laughs), so that should be handled in a way that doesn't… because nowadays it feels like we're blamed for everything bad, but no, we're not— the barriers are set against us when we… I don't want any medals, but don't blame me either, I know exercise is good, if someone falls, it's an accident, it's like if I prescribe aspirin, and the person turns out to be allergic, and I didn't know, well… people tend to think "damn, the doctor told me to take the stairs, and look, I fell and hurt my knee", you know? I don't know if I'm explaining myself" (13MGPUrb).
	Emotional Harm (Shame)	"But of course, the problem is that when patients don't recognize their physical inactivity, they mention daily tasks such as walking to do the shopping or taking the stairs instead of using the lift to justify, reflecting a certain degree of embarrassment. Others report having pain or discomfort when they walk a little, turning into a vicious cycle —weight, exercise, pain — and it's complicated" (06MGPUrb).
Justice	Fairness, equity and access	"In rural areas, people find it easier to go out for walks, maybe in the countryside, in open spaces, to go hiking or just to walk all around the village, and through the surrounding areas, along paths and trails and so on, whereas in the city that might be more complicated, but people there have better access to sports facilities such as swimming pools, gyms, athletics tracks, paddle or tennis courts, I think it's different, in rural areas it's usually older people, and that's the kind of exercise they do — going out for walks, and perhaps young people there don't have as many gyms or sports facilities that fit their interests, and in urban areas it's the opposite — there may be more options for younger people and so on, and maybe older people can always walk around the city, through parks and such, but… it might be more appealing for older people to walk in rural settings, along trails, or around the village" (03FnurseRur).
Emotional barriers to ethical practice	Professionals' frustration	"Courses are being offered continuously by the Training Commission, but many times you don't have access to those courses, because they're restricted, because you're not allowed to attend, you request permission but it's not granted, either because the course is full or due to service needs, and that's it, or sometimes, you know what also happens? that we can't even apply for them, because if you do, they don't assign a substitute for you, so your colleague has to take over your consultations and that makes you feel really guilty, you think, "If I go to take a course to improve my training, my colleague next door will have to handle my patients" so that often holds me back a lot from taking courses" (07FnurseRur).
	Role Modelling	"Observational learning is very important, I mean, I've seen it — because, as I told you, I've been working for many years, and I've seen doctors tell patients to stop smoking while holding a cigar in their own hand, yes, yes, yes, or, for example, say "you need to exercise," and it's being said by professionals who are obese, and some can even give you a masterclass explaining that their obesity is genetic, you know? Unfortunately, there are quite a few cases like that, maybe some of them really do have a genetic problem, you know what I mean, but still, no, if a person is talking to a patient about losing weight, and the patient has seen them for years and they haven't lost a gram themselves, you have to set an example" (01FnurseUrb).
		"Well, the time we're away our colleague has to cover for us, because you leave your patient list unattended, and during your absence, there may be needs, so we have to take things off our workload, because if health education is done in groups, it first requires time to prepare, at home to train yourself, and it also requires support materials,

Lack of support	computers, and so on, and it’s true that in that regard, the management doesn’t give us any support, meaning that if you want to run a group session, you have to do it on your own time, at home, outside of working hours, because during work hours, there’s simply no time, and even if you wanted to do it during work hours, the computer system we have isn’t effective, it doesn’t allow it, you can’t even make a PowerPoint presentation on the work computer, there are no IT resources, you have to use everything from home” (07FnurseRur).
