# Supplementary material for: Physical Activity Prescription in Primary Health Care: An Ethical Analysis
Source: Healthcare (Basel). 2026 Apr 3;14(7):934. doi: 10.3390/healthcare14070934 (PMC13073350; doi:10.3390/healthcare14070934)
Supplement: Supplementary file 1 [file healthcare-14-00934-s001.zip › healthcare-4179968 Table S2. Expanded Verbatims for Theme 2.pdf]

Table S2. Expanded Verbatims for Theme 2: Coping Strategies used to navigate ethical conflicts.

Sub-themes	Codes	Verbatims
Patient-centered ethical practice	Tailored assessment	"The method consists of explaining to the patient the benefits it has, but also how they should do it, that is, how to perform the exercise, it's like giving them a set of guidelines, exercise instructions, so to speak, going deeper into the tasks or into the treatment you prescribe, making that prescription detailed, right? Detailed, seeing what they can do each day, which exercises they should do daily according to their abilities and the needs of their specific condition, then you have to evaluate what they've done, schedule a review of that treatment to see if it's been effective and whether they've followed it or not, so basically it's about using physical exercise while giving it the importance it truly deserves" (14FGPUrb).
	Sustainability	"It has to be sustainable, meaning absolutely compatible with the person's life and condition — their physical condition, whether they have any illness or health problems — but also with their job, and with their social and family life. Just like a diet, exercise must be prescribed taking all of this into account, and a person has to find that balance — the right kind of exercise, at the right time, in the right way, and at the right intensity — so that it becomes integrated into their life, that's the main goal: for it to become part of their life, for it to be sustainable, because exercise is for life, it's not something prescribed for one month or three months, exercise is a lifestyle habit, it sustains health for as long as it's practiced — if you stop, it stops sustaining it" (05MnurseRur).
	Active listening	"That reflection the patient makes about what you propose is very motivating because in that moment you're encouraging them to make decisions and choose for themselves, and sometimes it really helps to give them several options… so they can choose it's like you make them an active participant in their treatment plan you make the treatment more tailored to them, to their preferences, right? to their… and that's basically how I do it" (16FnurseRur).
	Inclusive options	"You have to find the patient's motivation, it shouldn't feel like an obligation, it should bring some enjoyment, because for there to be adherence, the patient has to find sustainability and part of that sustainability is that it shouldn't feel like a sacrifice, they should develop a habit, but not one that feels burdensome, they should even be able to enjoy that physical activity — whether it's with someone, outdoors, or doing whatever they find interesting, whether it's dancing or swimming, whatever they enjoy, otherwise, there's no adherence, if not, they get bored and end up quitting, it has to be enjoyable, it has to be enjoyable and… that's the way to make it work, it has to be done in a planned and methodical way so that it becomes a habit, otherwise, it doesn't become sustainable" (05MnurseRur).
	Explanation of purpose and risks	"Well, here's something important, and it's very important what I'm about to say, and you know it, not only so that it's effective, but also so that it isn't harmful, because many times the way exercise is prescribed ends up being counterproductive, you have to look at the person as a whole, for example, someone who has knee problems but also high blood pressure or high blood sugar — if you tell them to walk for an hour, you'll end up damaging their knees, right? you can't make a prescription without taking into account all their conditions, everything that person is, in their full context — not just the physical or pathological aspects, but also their social and family context, it has to be compatible with their life, compatible with their job, otherwise it leads to rejection or they end up thinking exercise isn't good, or they abandon it for countless reasons because it doesn't fit into their life or their reality — whether physical, social, work-related, or family-related" (05MnurseRur).
		"To do follow-up, because otherwise, as I say, the healthy advice you give is useless, because if you give advice or prescribe something but then don't know whether the patient is actually doing it, it really serves no purpose… I mean, ideally, there

Ethical continuity and accountability	Follow-up & documentation	should be a follow-up to see whether it's being done, because in the end, it's a prescription for something, and you want to achieve a goal, so to know whether that goal has been achieved, you need to follow up and re-evaluate the patient" (18FnurseUrb).
	Structured planning	"Maybe we should focus more on also giving the patient a structured physical activity plan, on actually prescribing it, agreeing with the patient on goals that are realistic and appropriate and, above all, on assessing how the patient perceives that physical activity prescription, whether they see it as something valuable, whether they consider it important, whether they follow it, and whether they seek any kind of guidance or believe that the activity they already do is sufficient" (20MnurseUrb).
Professional identity and credibility	Personal experience	"Your personal perception of physical exercise has a big influence on how you convey it to the patient, I mean… and maybe it sounds harsh, but… if you come across as an unhealthy person, someone who doesn't take care of themselves, who is overweight, how can you tell a patient to lose weight? if you're the first one who should lose it, I mean, it might sound a bit blunt… but yes, in that sense, the credibility of your recommendation is somewhat called into question, and honestly, I don't know how that could be translated into an area for improvement, I really don't, but it's true that depending on what you think about exercise and how much you practice it and believe in it yourself the patient will believe in it too" (16FnurseRur).
	Professional recognition	"I proposed to the manager a form of social reinforcement — "what can we do? let's organize a public event where you call up the person who's kept the best records, the one who's done the most health education" so we held an event where we simply printed out from the computer a certificate in which the manager thanked them and acknowledged their contribution to the system, and so on, and their colleagues applauded them as they went up to receive it, and look — they left saying, "I feel like coming back to work tomorrow to keep doing it, because I'm sure I can do even better", just imagine the impact — the effect that social reinforcement can have" (01FnurseUrb).
Shared ethical responsibility	Interprofessional collaboration	"Well, in principle, if it's in primary care, it would involve relatively basic physical activity, the physician could make the prescription and the nurse could monitor the program every 15 days or every three weeks, depending on the type of patient and the condition or pathology being treated with physical exercise, that would be the kind of system that could make this a truly successful treatment, and then, fortunately, there are sports where the athletic lifestyle is increasingly valued socially — it's a paradox that obesity keeps rising, yet the athlete's profile is becoming more and more admired" (02MGPRur).
